# A strategy for extracting and analyzing large-scale quantitative epistatic interaction data

**DOI:** 10.1186/gb-2006-7-7-r63

**Published:** 2006-07-21

**Authors:** Sean R Collins, Maya Schuldiner, Nevan J Krogan, Jonathan S Weissman

**Affiliations:** 1 Howard Hughes Medical Institute, Department of Cellular and Molecular Pharmacology, University of California-San Francisco and California Institute for Quantitative Biomedical Research, San Francisco, California 94143, USA; 2 Banting and Best Department of Medical Research, University of Toronto, College Street, Toronto, Ontario, Canada M5G 1L6; 3 Department of Medical Genetics and Microbiology, University of Toronto, Kings College Circle, Toronto ON, Canada M5S 1A8; 4 Department of Cellular and Molecular Pharmacology, University of California, San Francisco, San Francisco, CA 94143, USA

## Abstract

Recently, approaches have been developed for high-throughput identification of synthetic sick/lethal gene pairs. However, these are only a specific example of the broader phenomenon of epistasis, wherein the presence of one mutation modulates the phenotype of another. We present analysis techniques for generating high-confidence quantitative epistasis scores from measurements made using synthetic genetic array and epistatic miniarray profile (E-MAP) technology, as well as several tools for higher-level analysis of the resulting data that are greatly enhanced by the quantitative score and detection of alleviating interactions.

## Abstract 2

A new technique for analysis of data from synthetic genetic array and E-MAP technology generates high confidence quantitative epistasis scores.

## Background

Genetic (or epistatic) interactions, which describe the extent to which a mutation in one gene modulates the phenotype associated with altering a second gene, have long been used as a tool to investigate the relationship between pairs of genes participating in common or compensatory biological pathways [1,2]. Recently, it has become possible to expand the study of genetic interactions to a genomic scale [3-7], and these new approaches provide a previously unseen perspective of the functional organization of a cell. The structure of this network of genetic interactions contains information that will be critical for understanding cellular function, the interplay between genotypes and drug efficacy, as well as aspects of the process of evolution, such as the maintenance of sexual reproduction [8,9].

Formally, genetic interactions can be defined in terms of deviation (ε) from the expectation that the combined effect on the fitness of an organism of two mutations will be the product of their individual effects:

ε = W_ab _- W_a_W_b _    (1)

where W_a_, W_b_, and W_ab _represent the fitnesses (or growth rates) relative to wild-type organisms with mutation A, with mutation B, and with both mutations, respectively. Non-interacting gene pairs have ε close to zero, synthetic sick and synthetic lethal (or synergistic) pairs have ε less than zero, and alleviating (or antagonistic) gene pairs have ε greater than zero [8]. A number of studies indicate that ε is typically close to zero, although the generality of this suggestion remains to be established [9,10]. More broadly, however, it is clear that the phenotypes associated with each individual mutation must be considered when evaluating the phenotype of the double mutant. Indeed, a double mutant could have a more severe phenotype than either single mutant and still represent a synthetic, neutral, or alleviating interaction. Typically, large-scale studies have scored gene-gene interactions in a binary manner (synthetic sick/lethal or noninteracting) [3,4,6,7]; however, synthetic lethal interactions are only one extreme example of a much broader phenomenon [9,11]. A binary score will then sacrifice information on the strength of interactions, as well as the entire notion of alleviating interactions.

Genetic interaction data can, in principle, be gathered in any of a number of ways. In practice, two large-scale techniques have been effectively executed in yeast. One, the synthetic genetic array (SGA) method, uses a set of selectable markers and several rounds of selection following the mating of one mutant strain with one marker to an entire library of yeast deletion strains with a second marker to recover haploid double mutant strains systematically and in large-scale. Sizes of colonies of double and single mutant strains grown for a defined period of time after transfer of a defined number of cells are then measured in high-throughput [4,6,12]. The other technique, termed diploid synthetic lethality analysis by microarray (dSLAM), uses deletion strains containing molecular barcodes and a microarray detection technique to measure relative growth rates of mutant yeast strains in competition [3,7]. In order to study smaller, rationally designed subsets of the genome, a variation of the SGA method, termed epistatic miniarry profile (E-MAP), was developed and used in the work analyzed here [5]. In E-MAP experiments, a rationally chosen subset of the genome is studied, and all genetic interactions between pairs of genes in this subset are measured.

We present here, and make freely available online [13,14], an integrated set of analytical strategies for processing raw colony array images from E-MAP [5] and SGA experiments to extract reproducible, quantitative measures of epistasis. Our analytical strategies were developed in parallel to the creation and study of E-MAP data for the early secretory pathway (ESP) in *Saccharomyces cerevisiae *[5], and these data were used as a test for our methods. We are presently applying our methods to additional logically selected subsets of genes; however, all results presented in this paper arise from analysis of the ESP data. E-MAP experiments intrinsically include two measurements of each genetic interaction based on distinct constructions of each mutant strain, and so from our measurements we can compute intrinsic estimates of measurement error and provide a natural estimate of the confidence with which genetic interactions can be assigned. In addition, we develop techniques and algorithms for using these quantitative epistasis measurements to derive detailed information about the functional relationships between pairs of genes, the general functional process a gene participates in, and the relationships between distinct functional processes within a cell.

## Results and discussion

### Processing raw SGA data

**Figure 1:**
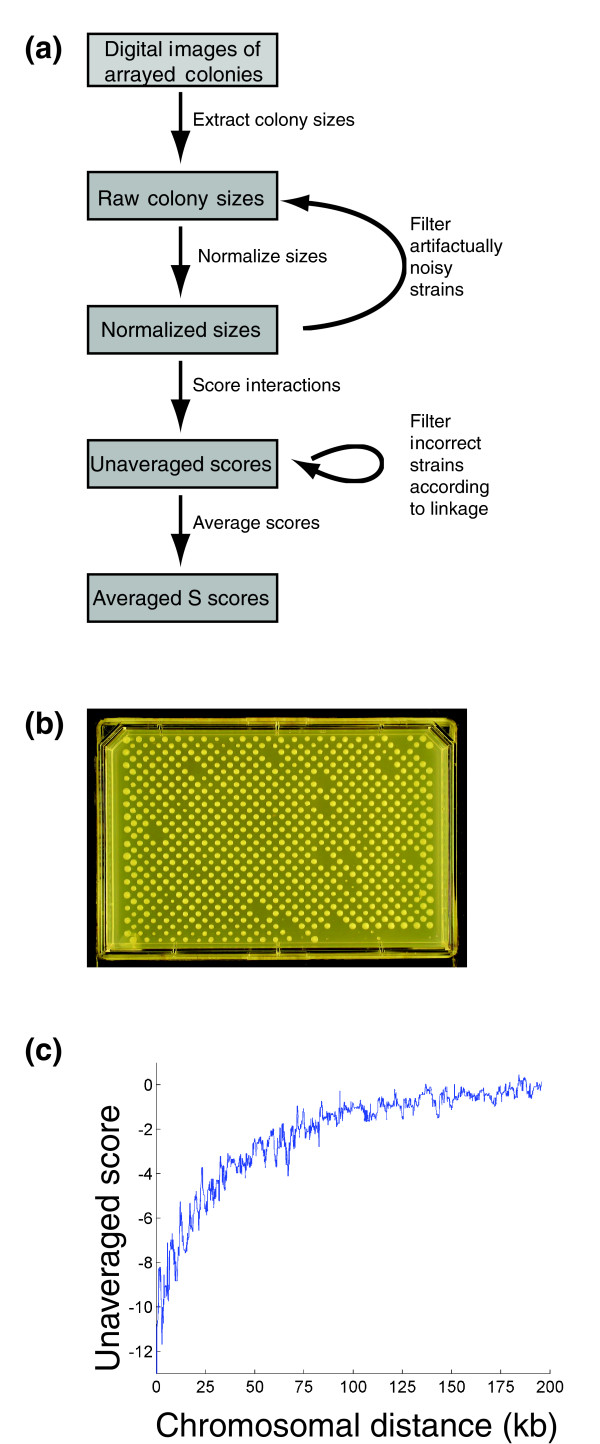
Overview of scoring procedure. **(a) **Schematic of the procedure used for generating interaction scores from jpeg images of double mutant yeast strain colonies. **(b) **A representative image of colonies of haploid double mutant yeast strains arising from the mating of one NAT-marked mutant strain to an array of 384 KAN-marked mutant strains, followed by sporulation and a selection process. **(c) **Median interaction scores as a function of the distance in kilobases between genes. All analysis shown is performed on data from Schuldiner *et al*. [5].

The utility of large-scale interaction data sets is highly dependent on the confidence that can be assigned to their results. Additionally, gene-gene interaction measurements have typically been scored as all or nothing phenomena, while, in fact, a continuum of genetic interaction strengths exists. The extra information contained in the varying strengths of genetic interactions may be extremely useful for teasing apart the organizational structure of the cell and for determining gene functions. In fact, efforts to take advantage of the quantitative nature of chemical-gene interactions have already proven useful [15-17]. We present here a new method for the processing and error-correcting of data from one large-scale genetic interaction measurement technique, the SGA method and its variation (E-MAP). The strategy can be visualized using a flow-chart (Figure 1a). Our data processing results in significantly lower error rates and more quantitative data than previous implementations of SGA techniques, and, specifically, it produces more reproducible scores than a standard t-test scoring of genetic interactions using the same raw data (see below).

In SGA experiments and in the E-MAP experiments analyzed here, double deletion strains are made systematically by crossing a query strain, defined as a strain with one genetic modification (for example, a gene deletion) marked with a gene for resistance to Nourseothricin (NAT), against a library of (in this case 384) test strains, each carrying a unique genetic modification marked with a gene for kanamycin (KAN) resistance. Through an iterative selection process [4,6,12], a haploid strain is obtained for each pair of mutations. During the selection process, haploid strains derived from crosses between query and test strains are grown on single selection media and the double mutants compete directly against single mutants. Finally, all 384 double mutant strains arising from an individual query strain are grown simultaneously on the same plate under double selection, and growth is quantified by the measurement of colony areas after a defined period of time (Figure 1b; see Materials and methods) [12]. One then would like to convert these colony areas into scores that represent the fitness of a double mutant relative to the fitness that would be expected given the fitnesses of each single mutant. These scores should be able to discriminate both synthetic genetic interactions, where double mutants grow more slowly than expected, and alleviating interactions, where double mutants grow more rapidly. Previous experimental and theoretical work indicates that the expected growth phenotype should depend on the phenotypes of each single mutant [8-10]. Importantly, this expectation means that the growth phenotypes of double mutants must be doubly normalized, to account for the growth defects associated with each single mutation, in order to score genetic interactions accurately. Additionally, measurement error must be carefully considered to distinguish real genetic interactions from simple experimental variability.

The first normalization is simple: colony sizes on each plate are scaled according to the typical size of a colony on the plate (see Materials and methods). This normalization accounts for growth defects associated with the query strain, as well as for differences in growth conditions from one plate to the next.

The second normalization or correction must account for growth defects directly associated with each test strain. Previously, these growth effects were accounted for by comparing the areas of double mutant colonies to the areas of colonies generated from control screens in which an appropriately marked wild-type strain was used as the query strain. While this strategy should in principle be effective, we found that errors in the measurement of the control colony areas created systematic biases that affected all double mutants arising from particular test strains (that is, all double mutants carrying a particular KAN-marked mutation).

We therefore adopted an alternative scoring strategy that takes advantage of the fact that genetic interactions are rare [3-5]. We used the median of the colony sizes, normalized to account for the effect of the mutation in the query strains, of all double mutants arising from the same test strain as our control. These values were highly accurate, since they represent the median of a very large number of measurements, and obtaining them requires no extra labor. Systematic errors were limited because all strains used for comparisons were grown under the same conditions, and because each double mutant was constructed twice (once with each possible query strain), correcting for any asymmetries in our scoring procedure. Most importantly, this score allowed us to measure both synthetic and alleviating interactions, as both would have colony sizes differing from the control value.

In addition to estimating an expected size for each double mutant, we also needed to estimate measurement variability in order to create a reliable score. Each double mutant colony size was measured in six replicates (two duplicate measurements on each of three independent experimental plates), allowing a natural measure of variation in the standard deviation. However, the standard deviation is only an estimate of experimental variability, and, with a relatively small number of measurements, it can be a significant source of noise. In particular, measurements with an unusually small standard deviation would result in scores of increased magnitude, even though they would not correspond to stronger phenotypes. For this reason, we took a reliable, though conservative, dual approach for estimating experimental error by including a minimum bound based on the average of the standard deviation for many similar double mutants (Additional data file 1). This strategy is conceptually similar to an approach taken for the analysis of microarray data in which Bayesian estimates of experimental error were used rather than the measured standard deviations [18]. The dual strategy provided very accurate estimates of variability while still detecting noisy, less reliable individual experiments, and empirically it led to a stronger correlation between scores for identical gene pairs over duplicate measurements (see below).

Interaction scores (S scores) were then calculated for each pair of genes using a modification of the t-value equation that included our own calculated expected colony size and corrected variances (see Materials and methods for equations). It is important to note that this score may not in general be equivalent to the epsilon value defined in equation 1, as it may be sensitive to both effects on logarithmic growth and on saturation of growth, nor does it rest on the assumption that such an epsilon is typically close to zero.

### Quality control

We took advantage of the experimental design to add critical quality control steps. Because mutations of two genes located on the same chromosome could only segregate to the same spore if a recombination event occurred, double mutations of gene pairs with low recombination frequencies resulted in negative S scores (Figure 1c). We could therefore check if our markers had been integrated at the correct chromosomal locations by examining the S scores of double mutations of neighboring genes. Of particular use were crosses of query and test strains with the same mutation, since in this extreme case the recombination frequency between the markers should always be zero. Using this analysis (see Materials and methods), we discovered that approximately 11% of our original libraries consisted of incorrect strains (see Additional data file 2 for a list of the removed strains and Schuldiner *et al*. [5] for a list of all strains used in the study). It is not clear what fraction of these strains was incorrect in the original libraries and what fraction became corrupted during the course of the experiment. Incorrect strains were removed from the data set, and when possible remade and remeasured to generate replacement data. Additionally, all scores for gene pairs with chromosomal locations within 50 kb of each other were removed from the data, as these scores would tend to be negative whether or not a synthetic genetic interaction exists between the two genes (Figure 1c).

**Figure 2:**
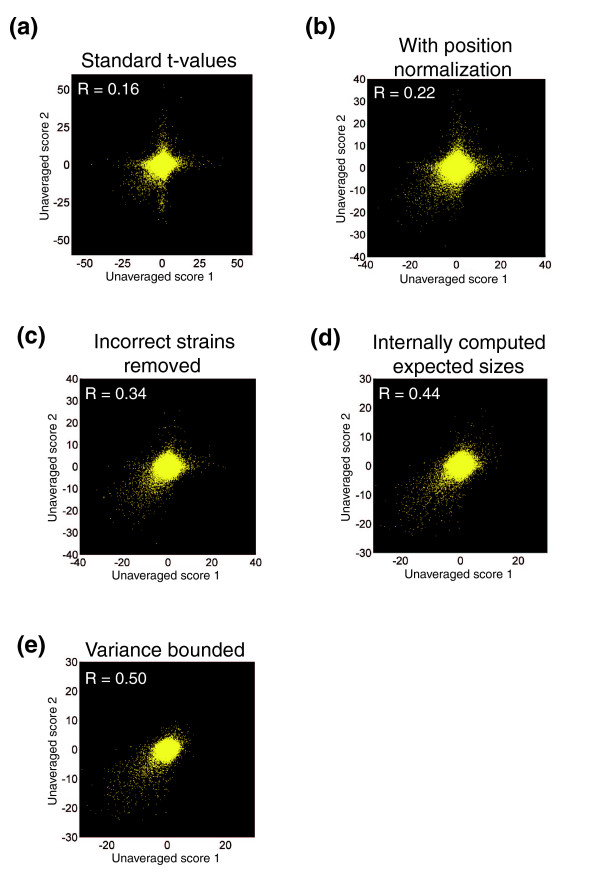
Effects of modifications in the S score (from a t-value) on score reproducibility. Each panel contains a scatter plot, in which each point represents two independent score measurements for a single pair of genes. All panels use the same raw data, but they differ in the scoring procedure used. The two scores come from the two possible pairings of antibiotic resistance markers with gene mutations. **(a) **Standard t-values in which colony sizes are normalized according to the mean colony size on the experimental plate. **(b) **Standard t-values using the normalization procedure described here. **(c) **Standard t-values with the normalizations described here and the removal of incorrect strains and experiments. **(d) **S scores without minimum bounds on variances. **(e) **Full S scores.

Additionally, large standard deviation measurements were used to identify unusually noisy test and query strains, which likely resulted from contaminations or technical errors in the plating process. A decision to remove or keep these strains was then made after visual inspection of the raw images. To prevent user bias, this inspection of images was done in blinded fashion. A significant number of such strains were identified and removed, and the scoring process was repeated. These scores, which included steps to account for and minimize the effects of experimental noise as well as extensive quality control, were markedly more reproducible than a scoring of the same raw data using a standard t-value (Figure 2). Each of the above described steps contributed significantly to the improvement in score reproducibility (Figure 2). The standard t-value scoring arises from the standard t-value calculation using the means and variances of normalized double and single mutant colony sizes (see Materials and methods for equation).

Finally, all measurements corresponding to the same gene pair were averaged to create one composite score. For gene pairs with only one measurement, a pseudo-averaging was performed to obtain the most likely averaged score, given the single score (see Materials and methods). The pseudo-averaging was included because, particularly for noninteracting gene pairs, averaging tends to result in scores of smaller magnitudes, and we did not want to place more weight (in the form of larger magnitudes) on scores for which less data were collected.

### Assessing data quality

**Figure 3:**
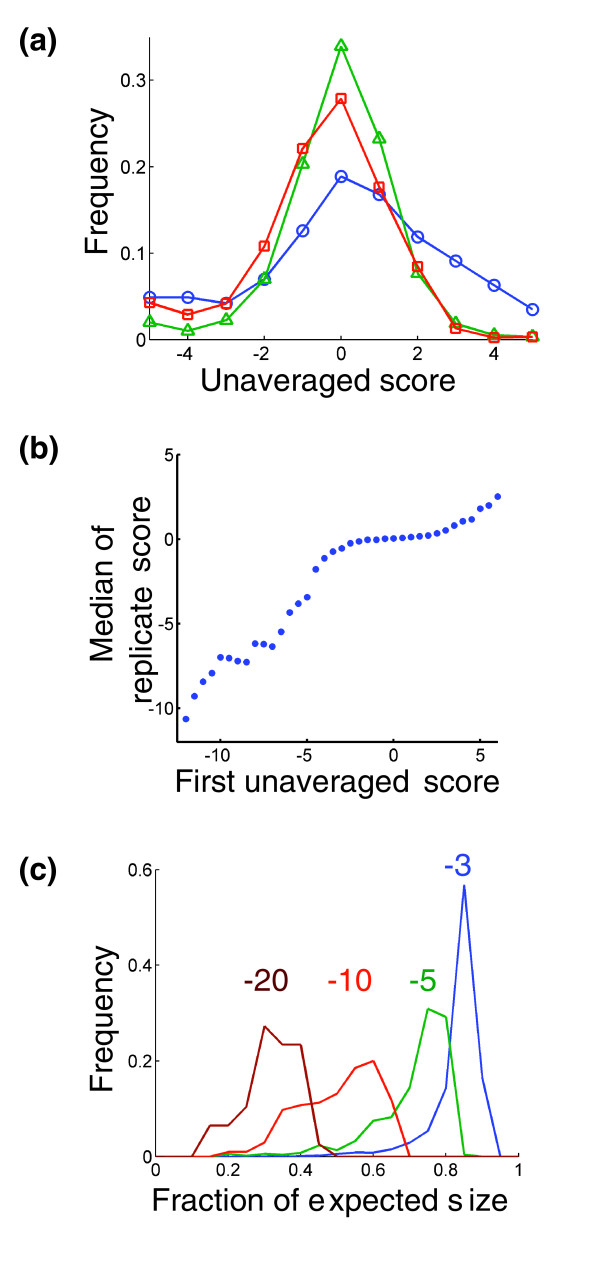
S scores are unbiased and quantitative. **(a) **Distribution of S scores for pairs of genes whose individual mutations give different growth phenotypes. The curves represent scores from pairs of genes whose individual mutations both yield slow growth phenotypes (blue circles), both yield growth phenotypes typical of our set of mutant strains (green triangles), and both yield relatively fast growth phenotypes (red squares). **(b) **Median interaction score on the second measurement (from an independent construction of strains) for pairs of genes with the indicated score on the first measurement. **(c) **Histograms of the observed colony size divided by the expected colony size for double mutant strains with S scores of approximately -3 (blue), -5 (green), -10 (red), and -20 (brown).

We assessed the quality of the data set with several goals in mind. First and importantly, we found that our scoring system displayed no systematic bias due to the phenotypes associated with individual mutations. The most common S score was zero, even when both mutations were associated with large or small colony size phenotypes (Figure 3a). This result was not guaranteed by our selection of the scoring system, and it provides independent validation that our multiplicative normalization worked well over the full range of mutant phenotypes observed, allowing accurate detection of the lack of genetic interaction in a typical double mutant. Additionally, we wanted to determine the degree of detail we should be able to extract from our scores with respect to two significant considerations. We wanted to understand whether the genetic interactions we observed gave us quantitative or qualitative information, and to characterize the confidence with which we could assign genetic interactions.

To assess whether quantitative information was contained in the S scores, we took advantage of the fact that each double mutation strain was constructed twice - once with each of the two possible query strains. We found that scores close to zero, which should be indicative of no genetic interaction, typically repeated as scores close to zero in the second measurement. For these scores, there was little correlation between the first and second scores. However, for scores of magnitude greater than approximately 3, the first score was highly predictive of the second score with a near-linear relationship (Figure 3b). Furthermore, by reexamining our colony size measurements, we confirmed that variations in the magnitude of negatives scores indeed correspond to differences in the relative fitness of the double mutant strains (Figure 3c). Formally, these variations in score could have also been due to differences in expected colony sizes and measurement variabilities.

**Figure 4:**
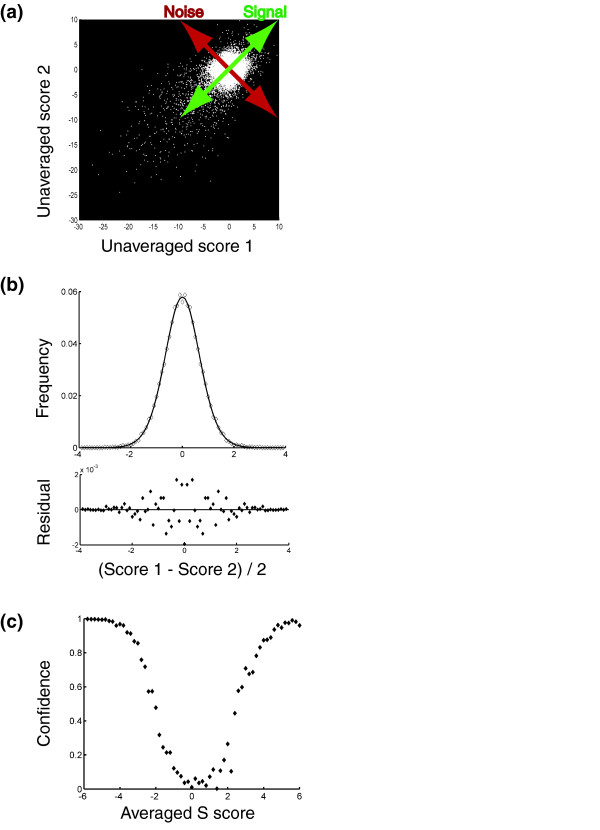
Estimating significance for S scores. **(a) **Schematic illustrating the strategy used to estimate the distribution of S scores arising from noninteracting gene pairs. The distribution of pairs of scores lying close to the 'Noise' axis (that is, pairs with an average score close to zero) were assumed to arise from noninteracting gene pairs. **(b) **Fit (with residuals shown below) of the distribution of scores lying close to the 'Noise' axis in (a) according to the model that individual S scores for noninteracting gene pairs follow a t-distribution (see Materials and methods for further explanation). **(c) **Plot of an estimate of the fraction of observations, as a function of averaged S score, that correspond to genuine genetic interactions.

We were further able to use the intrinsic redundancy in the data set to estimate a confidence level that any given averaged S score represents a significant interaction. The confidence values were obtained by computing an estimate of the distribution of scores that arise from noninteracting gene pairs (Figure 4a,b; see Materials and methods). With the distribution of scores from noninteracting pairs and the total distribution of scores, we could then estimate the fraction of observations, for each given averaged S score, that correspond to real interactions (Figure 4c). Although this method does not account for all potential sources of systematic error, it does account very well for measurement variability and some systematic errors. Importantly, an experimental validation of interactions for *IRE1 *and *HAC1*, which mediate an endoplasmic reticulum specific stress response termed the unfolded protein response (UPR), independently established the validity of interactions judged to be significant [5].

### Extracting functional information

**Figure 5:**
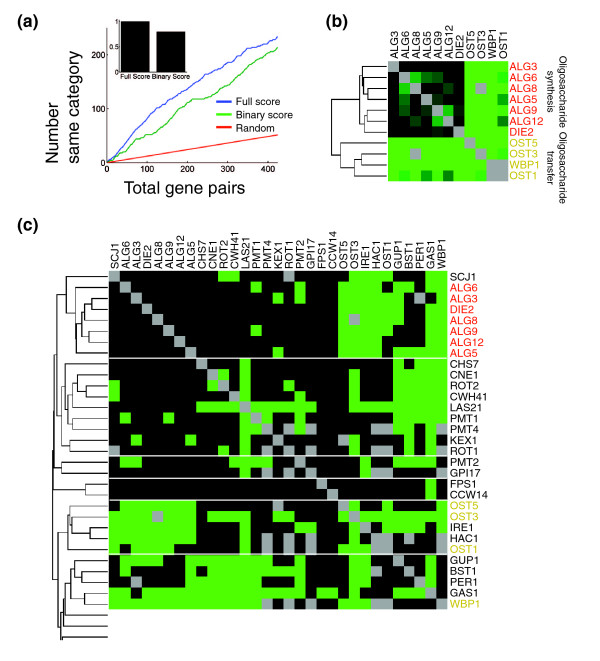
Quantitative genetic interaction scores allow for more precise functional characterization than binary scores do. **(a) **Different methods for scoring genetic interactions were compared by their propensity to yield high correlations between the profiles of pairs of genes that were assigned to the same functional category. For the S scores and for a binary score in which interactions were classified as either synthetic or noninteracting according to a threshold in the S score, gene pairs were sorted from highest correlation to lowest correlation. The curves show the cumulative number of gene pairs belonging to the same functional category versus the total number of gene pairs for the full S score (blue) and for the binary score (green). The red curve indicates the expected result if the gene pairs are sorted randomly. Inset is a bar graph showing integrations of the two curves over the full range of gene pairs after subtracting the background of the random expectation. The threshold for the binary score was chosen to maximize the integral shown in the inset. **(b) **The minimal cluster including the seven ALG genes and the four OST genes when clusters are made using hierarchal clustering with the correlation between the profiles of S scores used as the metric. **(c) **The minimal set of clusters containing the ALG and OST genes when the same clustering algorithm is performed using the binary scores rather than the S scores.

Once accurate scores have been obtained, they can be used for higher order analyses. One common method is hierarchical clustering, which can be used, with each gene's profile of genetic interactions serving as a sophisticated high-dimensional phenotype, to gather much information about gene function. Analysis of the ESP E-MAP revealed that gene products functioning in highly similar processes can be identified solely by their similar patterns of genetic interactions, often with remarkable specificity and precision [5]. Importantly, and consistent with suggestions from studies of drug-gene interactions [17], we found that the quantitative nature of our score, as well as the ability to detect alleviating interactions, was critical for the success of clustering in accurately grouping related genes. Reducing our score to a binary score, in which gene pairs are classified as either synthetic sick/lethal or noninteracting, resulted in a decreased tendency for gene pairs that act in similar processes (as determined *a priori *by surveying the literature) to have highly correlated patterns of interaction (Figure 5a). This loss of resolution was also evident in the results of hierarchical clustering. For example, the ALG genes, which are involved in oligosaccharide synthesis, and the closely related OST genes, which function in the transfer of the resulting sugars onto proteins [19], are clustered together and neatly divided into their two natural subclasses using the full S scores, but when a binary thresholded score is used instead, they are split into several separate noncontiguous clusters (Figure 5b,c).

While hierarchical clustering proved very useful for illuminating gene functions, it also has a number of shortcomings. First, there were many proteins that did not fall into well-defined clusters. Second, there exist types of biological information in genetic interaction data that clustering is not suited to extract. For example, hierarchical clustering does not directly inform on the higher level organization of processes within the cell. Additionally, while clustering identifies proteins with similar functions, it does not resolve the specific relationship between these proteins. Therefore, new techniques tailored for detecting more complete and more precise biological detail could prove extremely informative. We present here several examples of such techniques, although many more are possible [10,20,21].

The already extensive annotation of the yeast genome, combined with the vast quantity of multidimensional data generated in large-scale genetic interaction experiments, presents an excellent opportunity for the use of supervised learning techniques to extract information that would otherwise have been inaccessible. We took advantage of these annotations both to create a method for examining the large-scale functional structure of genes in the ESP, and to generate high-quality predictions for the functions of many individual uncharacterized proteins. First, previously well-characterized genes in our data set were grouped into functional categories containing proteins that contribute to the execution of similar processes. This allowed us to measure the synthetic interactions within and between different functional processes by estimating p values for the enrichment of synthetic genetic interactions between pairs of categories. As might have been expected, we found that synthetic genetic interactions were often most commonly found between genes in the same functional category (for example, ER-Golgi traffic or lipid biosynthesis), but we were also able to identify pairs of distinct categories whose members are significantly more likely to interact than would have been expected by chance [5]. These enrichments of interactions between proteins in different processes can then be used to visualize the network of interdependencies between the different processes being carried out in an organelle or an organism [5].

Having patterns of interactions for each functional category also immediately provided us with a method of predicting the function of uncharacterized or poorly characterized proteins. We designed an algorithm that calculated a log p value for the enrichment of interactions between each gene and each category and compared the pattern of log p values for each gene to a similarly calculated pattern for each category [5]. The algorithm then predicted the functional category of a gene to be the category with the most similar pattern of interactions. We evaluated the accuracy of this method using 'leave-one-out' cross-validation [22] on the set of genes with assigned categories. Predictions were more accurate for genes with a substantial number of observed interactions and accuracy improved as the pattern for a gene better matched its most similar functional category. By setting minimum thresholds for these determinants such that predictions were made for 83 (50%) of the uncharacterized or poorly characterized proteins, we found that the algorithm performed at slightly better than 50% accuracy. Accuracy was noticeably better for proteins in the larger functional categories, and a sizeable fraction of the incorrect assignments were assignments to a similar category (for example, post Golgi traffic as opposed to intra Golgi traffic and vice versa). Several predictions for uncharacterized proteins were tested and confirmed [5].

**Figure 6:**
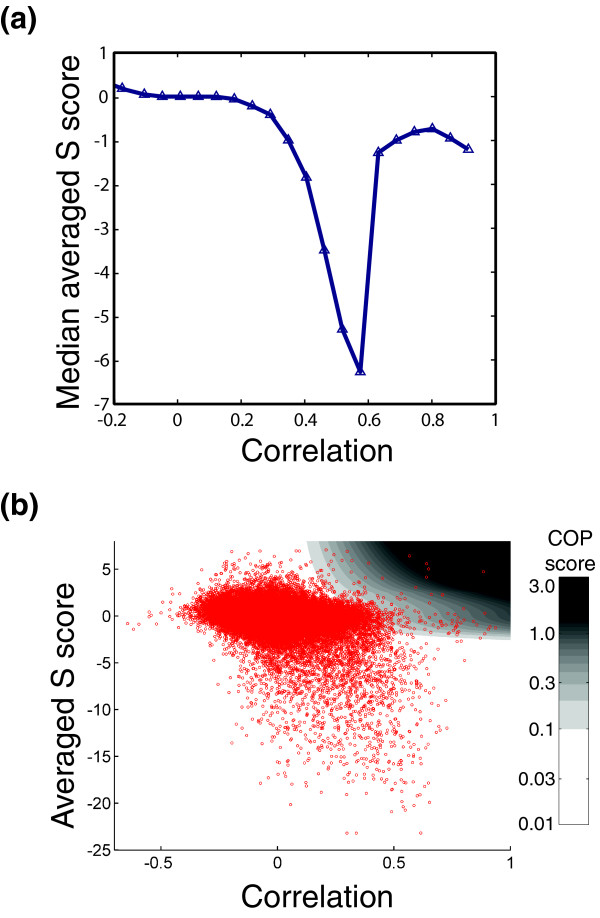
A score to identify coherently acting gene pairs. **(a) **Plot of median averaged S score as a function of the correlation between the patterns of genetic interactions for pairs of genes. Reprinted from Schuldiner *et al*. [5] with permission from Elsevier 2005 **(b) **Scatter plot of averaged S score versus correlation in which each point represents one pair of genes. The plot is overlaid onto a color gradient indicating the COP score.

Finally, careful analysis of genetic interaction scores can be used to pinpoint more specific relationships between proteins. To this end, we were motivated by two key considerations. The first is that if two genes have highly correlated profiles of genetic interactions, it indicates that they have similar functions, but it does not tell us how their functions are related. They could be in a physical complex or direct pathway, or they could be carrying out parallel or complimentary functions. The second observation is that a single genetic interaction, in the absence of further information, is extremely difficult to interpret. Therefore, we decided to look simultaneously at these two features, correlation and S score, to extract more information out of each of them. Although these features are mathematically independent, previous work suggested that genetic interaction networks tend to exhibit 'neighborhood clustering' where genes that interact synthetically with similar sets of partners are also likely to interact in a synthetic manner with each other [4]. Consistent with that observation, when we examined the median S score as a function of the correlation between interaction profiles, we found that highly correlated genes tended to exhibit synthetic interactions (Figure 6a). However, in striking contrast, the most highly correlated pairs of deletion mutations tended not to interact synthetically (Figure 6a) [5].

We reasoned that such high correlation and an alleviating interaction, or the lack of a measurable genetic interaction, is what would be expected of pairs of genes that function together in a direct linear pathway or in a dedicated protein complex. In such a case, the deletion of one gene could completely disable the complex or pathway making the second deletion essentially inconsequential. Therefore, we designed a score to identify such pairs (see Materials and methods). This score, called the COP score (for **CO**mplex or linear **P**athway), was rationally designed to identify gene pairs with a strong correlation between their profiles and a lack of a direct genetic interaction (Figure 6b; see Materials and methods). Many of the top hits were known protein complexes and direct pathway components, and we were also able to identify numerous other potential interactions, some of which were tested and confirmed with affinity-purification experiments [5]. Other, similarly motivated approaches are capable of giving similar results [21], and we hope that in the future, analysis of a larger data set including both genetic and physical interactions will allow optimization of a score using supervised learning.

## Conclusion

By taking advantage of the inherent redundancy in E-MAP data we were able to refine a qualitative binary scoring system into a quantitative system in which we could detect not only synthetic genetic interactions, but alleviating ones as well. As these interaction scores reflect real gradations in the relative fitness of double mutants, we find that genetic interactions occur in a spectrum of strengths and types. Furthermore, both the quantitative nature of the score and the detection of alleviating interactions were critical for the quality of higher level data analyses. We expect that the tools presented here should be useful for analysis of E-MAP and SGA data, and with fairly straightforward modification, they could also be applied to large-scale chemical-genetic studies.

## Materials and methods

### Brief overview of approach

Crosses and isolation of double mutant strains was done as previously described [12] with the modifications indicated below. A digital camera was used to obtain jpeg images of the resulting colonies using the setup described below. These images could then be converted to numerical arrays of colony areas using an executable Java program (see below). The output files of from this program are suitable to be read and analyzed using a MATLAB toolbox that implements all of our algorithms for the normalization, quality control, scoring, and confidence assessment of E-MAP data. The MATLAB toolbox is available for download at [14]. This download includes a pdf file with detailed instructions for its use.

### Data collection and image capturing

KAN-marked deletion strains were obtained from a preexisting library [23] and NAT-marked strains were constructed *de novo *[5]. Since the completion of this work, advances have been made in the protocol for *de novo *construction of the NAT-marked strains [24], and these advances may improve experimental accuracy in future studies. Synthetic genetic array technology was used in a high-density E-MAP format [5] essentially as described [12], except for the following exceptions. Manual pinning in 384-format was performed throughout the screen using manual pin tools (VP384F), library copiers (VP381) and colony copiers (VP380) from V & P Scientific, Inc. (San Diego, CA, USA). Only the final selection for double mutants was pinned robotically in a 768-format. The final double mutant plates were routinely grown for three days before pictures were taken using a set-up consisting of a KAISER RS 1 camera stand (product code-no. 5510) and a digital camera (Canon Powershot G2, 4.0 Megapixels) with illumination from two Testrite 16 × 24 Light Boxes (Freestyle Photographic Supplies product#1624) (see Additional data file 3 for an image of the setup). Images had a final resolution of 160 dots per centimeter. Initial spot areas from the pinning step were typically 20 pixels or smaller, and the final are of colonies in the images and were typically around 500 pixels.

### Image analysis

We have created and provide an executable Java program that identifies colonies arrayed in grid format and measures the corresponding areas. The output of this program is suitable for use with the MATLAB toolbox described below. The executable program can be downloaded from [13]. This download includes a pdf file containing instructions for the use of the program.

### Normalization of colony sizes

The sizes of colonies (areas measured in pixels) were normalized to correct for differences in growth conditions. The normalizations used here were multiplicative normalizations. We tried other normalization methods as well (including a logarithmic normalization) and found them to be less effective. Importantly, the normalization and scoring procedures presented here gave virtually identical results when the same experimental plates were imaged after different growth times (for example, after 2, 3, 4, or 5 days; data not shown). For each plate, a value referred to as the plate middle mean (PMM) was computed as the mean of the colony sizes ranked in the 40th to 60th percentile of colony sizes on the plate, excluding the outermost two rows and columns on the plate. The sizes of colonies in the outermost two rows and columns were then scaled such that the median size of each such row or column was equal to the PMM. The colonies in the outermost rows and columns were treated as a special case because their sizes tended to be noisier than the sizes of colonies in the center of the plate, and this extra variation tended to be consistent across each such row and column (that is, the entire top row might uniformly be unusually large or small). The sizes of colonies on the whole plate were then scaled such that the final PMM was equal to a fixed number (516.1) that was equal to the median size of all colonies measured in the study. That is:

(Normalized Size) = (Unnormalized Size) × 516.1/PMM

It should be noted that this normalization also corrects for differences in the growth phenotype typical of double deletions containing the NAT-marked mutation on each plate (that is, it corrects for any growth defect associated with the NAT-marked query strain). Additionally, a maximum normalized colony size of 1,000 was applied to minimize the effect of artifactually large colonies resulting from plate position, colony smearing, normalization of large colonies on plates with small PMM values, or other causes.

### Scoring genetic interactions

Double deletions were scored as to the magnitude and sign of the observed genetic interaction. We wanted a score that would reflect both our confidence in the presence of genetic interactions as well as the strengths of interactions, and so we chose to use a modified t-value score (S). A standard t-value is computed as:

t = (μ_Exp _- μ_Cont_)/sqrt(s_Var_/n_Exp _+ s_Var_/n_Cont_)

where:

s_Var _= (var_Exp _× (n_Exp _- 1) + var_Cont _× (n_Cont _- 1))/(n_Exp _+ n_Cont _- 2)

where: μ_Exp _= mean of normalized colony sizes for the double mutant of interest;

var_Exp _= the variance of the normalized colony sizes for the double mutant of interest; n_Exp _= number of measurements of colony sizes for the double mutant (typically, this value was 6, although it differed slightly (4, 10, and so on) for a small number of double mutants); μ_Cont _= mean of normalized colony sizes for the control KAN-marked single mutant strain corresponding to the double mutant of interest; var_Cont _= the variance of the normalized colony sizes for this control KAN-marked strain; and n_Cont _= the number of measurements of colony sizes for this control KAN-marked strain.

The S score is constructed in the same way:

S = (μ_Exp _- μ_Cont_)/sqrt(s_Var_/n_Exp _+ s_Var_/n_Cont_)

where:

s_Var _= (var_Exp _× (n_Exp _- 1) + var_Cont _× (n_Cont _- 1))/(n_Exp _+ n_Cont _- 2)

but with the following modifications: μ_Cont _= median of normalized colony sizes for all double mutants containing the KAN-marked mutant of interest; var_Exp _= the maximum of the variance of normalized colony sizes for the double mutant of interest or a minimum bound described below; var_Cont _= median of the variances in normalized colony sizes observed for all double mutants containing the KAN-marked mutant of interest or a minimum bound described below; and n_Cont _= 6 (this was the median number of experimental replicates over all the experiments).

#### Minimum bound on var_Exp_

A minimum bound was placed on the experimental standard deviation (and hence on the variance) because we observed that occasionally, by chance, six repeated measurements would give an unusually small standard deviation, resulting in a large score, but these large scores did not seem to be reproducible, nor did they reflect strong genetic interactions. We therefore placed a minimum bound on this standard deviation equal to the expected standard deviation in normalized colony size for a double mutant made from NAT- and KAN-marked mutants with similar growth phenotypes. The expected standard deviation was calculated by measuring the observed standard errors in measurement as a function of both unnormalized colony size typical of the NAT-marked mutant and as a function of normalized colony size typical of the KAN-marked mutant.

#### Minimum bound on var_Cont_

For similar reasons as for var_Exp _and because it improved the reproducibility of computed S scores, a lower bound was also placed on var_Cont_. This lower bound was equal to μ_Cont _multiplied by the observed median relative error (standard deviation divided by mean size) for all measurements in the data set.

#### Note concerning normalization using the PMM and scoring using the median size

Both of these measures may be biased if the frequency of synthetic interactions is significantly greater or smaller than the frequency of alleviating interactions for a particular gene. However, we have observed this bias to be relatively small (data not shown), and we include in our MATLAB toolbox an alternative strategy to estimate the typical colony size on an experimental plate or for a given KAN-marked mutant. The alternative strategy, which uses a Parzen Window [25] approach to estimate the most common colony size, is less sensitive to skewed distributions of colony sizes.

### Quality control

#### Removing unusually noisy experiments or strains

All query and test strains that gave rise to double mutant strains with a median or mean standard deviation of 80 (pixels) or greater were screened by eye to look for unusually noisy, incorrectly pinned, or otherwise unreliable experiments. These unreliable experiments were generally obvious, with either a substantial fraction of colonies missing or a complete lack of correspondence between the relative sizes of colonies among the duplicate experimental plates.

#### Removing strains based on linkage analysis

Closely linked pairs of genes (pairs that have a recombination frequency less than 50%) should give negative scores in SGA analysis because formation of spores carrying both markers will be disfavored (Figure 1b). Therefore, an algorithm was created and used to make a list of all deletions (NAT and KAN marked) in our library, and for each mutant, a sublist of all the double mutants containing the deletion of another gene within 40 kb, and the corresponding S scores. From this list, we manually determined which of the original single mutant strains in our original libraries did not have the marker integrated at the proper chromosomal location (and, therefore, were not the correct mutant strain). These incorrect strains could be identified by nonnegative S scores against mutations at nearby chromosomal locations. The incorrect strains (approximately 11% of the original libraries) were then removed from the data set and remade when possible (see Additional data file 2 for a list of the removed strains and see Schuldiner *et al*. [5] for a list of all strains used in the study).

### Creating the final data set

The final data set used for functional analysis consisted of a single score for each pair of genes (or a 'not a number' value (NaN) if no correct double mutant was made). This score was the arithmetic mean of all S scores for all separate constructions of the double mutant. In most cases this was an average of two S scores (*geneA::KAN geneB::NAT *and *geneA::NAT geneB::KAN*). In some cases (if multiple constructions of a single mutant strain were made), more than two scores were averaged, and in some cases only one measurement was made. In these cases, when no true averaging could be done, a pseudo-averaging was performed instead. This was done because unaveraged scores tend to have larger magnitudes than averaged scores, and we did not want to put extra weight on scores for which we had less data. The pseudo-averaging was done by averaging the observed score with the median second score observed for double mutants with a similar score on their first measurement (Figure 3b).

### Assessing confidence in genetic interactions

Our error analysis is based on the observation that S scores from noninteracting gene pairs are well-described by a t-distribution, with a width (σ) determined by the experimental noise (Figure 4b). We could estimate this distribution of scores in the following way: we assumed that pairs of scores close to the line of slope negative one passing through the origin (Figure 4a,b; that is, pairs of scores with an average close to zero) arose from these noninteracting gene pairs. By projecting these scores onto that line and normalizing the resulting distribution we obtained a probability distribution proportional to the square of the probability distribution of scores arising from noninteracting pairs of genes. Then by taking the square root of this distribution (point-by-point) and fitting the result to a t-distribution and normalizing it, we obtained an estimate of the probability distribution of single scores arising from noninteracting gene pairs. Next, the expected distribution of averaged S scores resulting from noninteracting gene pairs was obtained by convoluting this probability distribution with itself and normalizing the result to account for the number of gene pairs found in our original histogram. Subtracting the distribution of averaged S scores from noninteracting gene pairs from the total distribution of averaged S scores gives an estimate for the distribution of scores from interacting gene pairs. From the two distributions (scores arising from interacting gene pairs and scores arising from noninteracting gene pairs), we computed the approximate fraction of observations at each average S score that were due to real genetic interactions (Figure 4c).

### Mapping the patterns of interactions between functional categories

We estimated p values for enrichment of synthetic genetic interactions between all pairwise combinations of functional categories. These were computed using the formula:

P_ab _= ∑ (n!/i!(n - i)!) × p_ab_^i ^× (1 - p_ab_)^n-i^, i = x_ab_,... n

where: x_ab _= number of observed synthetic interactions between category a and category b; n = total number of observed synthetic interactions; p_ab _= (n_a_/n) × (n_b_/n); n_a _= number of observed synthetic interactions involving a protein in category a; and n_b _= number of observed synthetic interactions involving a protein in category b.

The base ten logarithms of these p values were then used to evaluate the degree of interaction between different functional categories.

### Predicting the functional category of individual genes

Functional categories were predicted based on the pattern of enrichment of interactions. Log_10 _p values for enrichment of interactions between a gene and a functional category were estimated using the formula:

P_gb _= ∑ (n!/i!(n - i)!) × p_gb_^i ^× (1 - p_gb_)^n-i^, i = x_gb_,... n

where: x_gb _= number of observed synthetic interactions between gene g and category b; n = total number of observed synthetic interactions; p_gb _= (n_g_/n) × (n_b_/n); n_g _= number of observed synthetic interactions involving a gene g; and n_b _= number of observed synthetic interactions involving a protein in category b. A synthetic interaction was considered to be significant with an averaged S score less than -2.5.

Similar calculations were done for each functional category and each gene for interactions with S score less than -6 (strong interactions). Two classes of genetic interactions (with cutoff values of -2.5 and -6) were used because empirically this improved predictive power. The cutoff values of -2.5 and -6 were chosen to roughly optimize predictive power, but small changes to these values (for example, -3 and -5.5) did not significantly alter results. These calculations gave a vector of log_10 _p values for each functional category and a similar vector for each gene. Prediction of functional category for each gene was done by finding the category whose vector had the highest (Pearson's) correlation to the gene's vector. We set the following thresholds for our predictions: genes must have at least two interactions (or one strong one) with genes in one of the functional categories being considered, and the correlation with the closest category must be at least 0.4. With these thresholds, predictions were made for 83 uncharacterized or poorly characterized genes. Accuracy of the predictions was estimated by using cross-validation [22] to test the accuracy of the prediction algorithm on genes already assigned to a functional category. Cross-validation was executed by sequentially removing each gene from our data set, recomputing all log_10 _p values for the categories, predicting the gene's functional category, and comparing this prediction to the gene's assigned category.

### Predicting membership in a dedicated physical complex or direct linear pathway

For each pair of genes, a COP score was computed. This score was rationally devised to identify pairs of genes that do not directly interact in a synthetic manner, but have strongly correlated patterns of genetic interactions. This score tended to identify pairs of genes in physical complexes and linear pathways. However, the set of genes in the study analyzed here contained only a small number of annotated physical complexes, and so we did not feel that this set was adequate for optimizing a score to identify complexes based solely on genetic interactions. However, we intend to pursue optimizing such a strategy using supervised learning techniques on a future data set containing both genetic and physical interaction data. The COP score used in this work was defined by:

COP = r^2 ^× (sc + 1) × mag

where: r = Pearson's correlation coefficient between the genetic interaction profiles of the two genes (for values of r less than zero, zero was used instead); sc = signed confidence that an interaction exists (this value is confidence value described in 'assessing confidence in genetic interactions' multiplied by the sign of the S score; so sc approaches -1 as an interaction is highly likely to be a real synthetic interaction and sc approaches 1 as an interaction is highly likely to be a real alleviating interaction - genes with correlated patterns and a synthetic interaction were thus automatically given a score of zero); mag = the minimum of 1 and the S score divided by 2.5 (this value is to give extra weight to strong alleviating interactions; since a score of 2.5 is approximately the point at which full confidence in an alleviating interaction is attained, this magnitude factor gives extra weight to scores that exceed this threshold in proportion to the score).

## Additional data files

The following additional data are available with the online version of this paper. Additional data file 1 is a scatter plot illustrating the application of a minimum bound correction for variances. Additional data file 2 lists the strains that were determined to be incorrect and were removed based on linkage analysis (described in text). Additional data file 3 is an image of the image capturing setup used for data collection. Additional data files 4, 5, 6, 7, 8 contain raw colony size data used for the analysis presented as formatted text documents. Additional data file 9 provides a short explanation of the data included in Additional data files 4, 5, 6, 7, 8. Final S scores and results are available at [26].

## Supplementary Material

Additional data file 1 Scatter plot illustrating the application of a minimum bound correction for variances. Click here for file

Additional data file 2 Strains that were determined to be incorrect and were removed based on linkage analysis. Click here for file

Additional data file 3 Image capturing setup used for data collection. Click here for file

Additional data file 4 Raw colony size data used for the analysis presented as formatted text documents. Click here for file

Additional data file 5 Raw colony size data used for the analysis presented as formatted text documents. Click here for file

Additional data file 6 Raw colony size data used for the analysis presented as formatted text documents. Click here for file

Additional data file 7 Raw colony size data used for the analysis presented as formatted text documents. Click here for file

Additional data file 8 Raw colony size data used for the analysis presented as formatted text documents. Click here for file

Additional data file 9 Explanation of the data included in Additional data files 4, 5, 6, 7, 8. Click here for file
