# Supplementary material for: A strategy for extracting and analyzing large-scale quantitative epistatic interaction data
Source: Genome Biol. 2006 Jul 21;7(7):R63. doi: 10.1186/gb-2006-7-7-r63 (PMC1779568; doi:10.1186/gb-2006-7-7-r63)
Supplement: Additional data file 1 — Scatter plot illustrating the application of a minimum bound correction for variances. [file gb-2006-7-7-r63-S1.pdf]

Additional File 1 - Minimum bound for variance

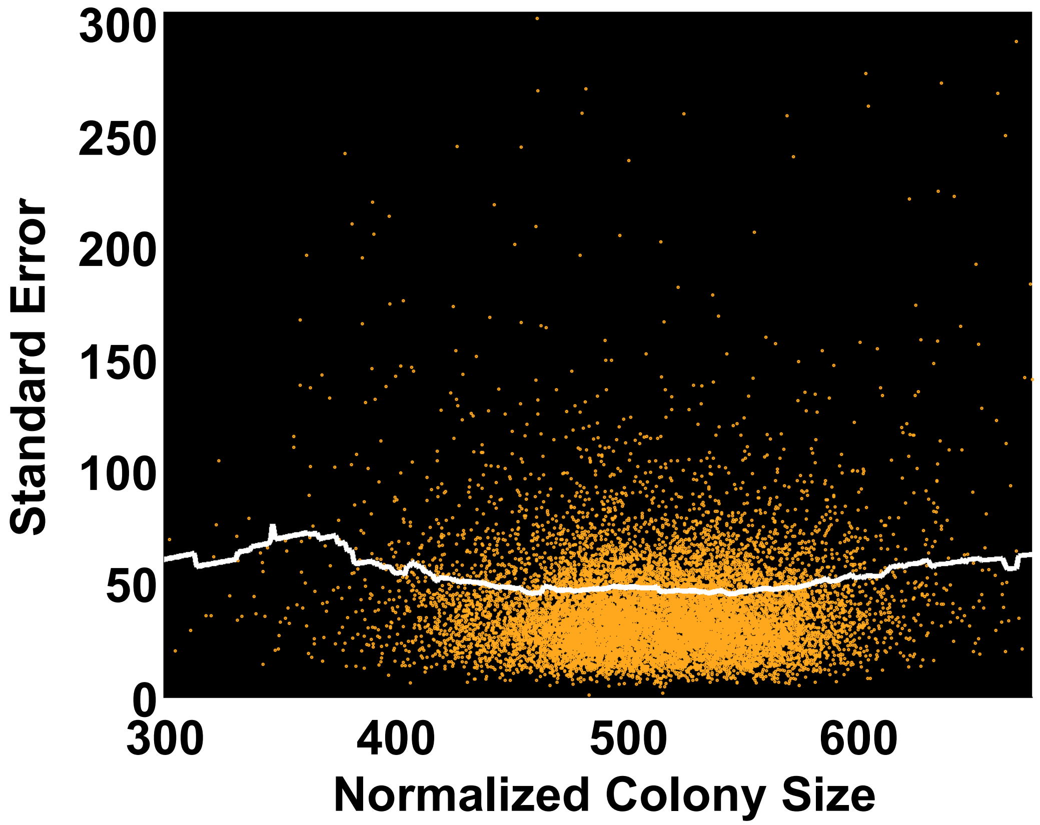

Use of a minimum bound on the measure of experimental variability. The standard deviations of measurements are plotted against the mean normalized colony size for colonies on plates with typical median sizes prior to normalization (490 to 510 pixels). In white is a curve of the minimum bound applied to the standard deviation (as a function of normalized colony size).
