# Supplementary material for: A strategy for extracting and analyzing large-scale quantitative epistatic interaction data
Source: Genome Biol. 2006 Jul 21;7(7):R63. doi: 10.1186/gb-2006-7-7-r63 (PMC1779568; doi:10.1186/gb-2006-7-7-r63)
Supplement: Additional data file 2 — Strains that were determined to be incorrect and were removed based on linkage analysis. [file gb-2006-7-7-r63-S2.doc]

| List of all strains that were removed from the data set on the basis of analysis of scores with closely linked genes | | | | | |
| --- | --- | --- | --- | --- | --- |
|  |  | |  |  |  |
| NAT-marked query strains: | | |  |  |  |
| Orf Name | | Gene Name | Marker | Mutation Type | Successfully remade? |
| YAL026C | | DRS2 | NAT | deletion | no |
| YBL017C | | PEP1 | NAT | deletion | yes |
| YBR106W | | PHO88 | NAT | deletion | no |
| YCR034W | | FEN1 | NAT | deletion | no |
| YCR044C | | PER1 | NAT | deletion | no |
| YCR067C | | SED4 | NAT | deletion | yes |
| YDL099W | | YDL099W | NAT | deletion | yes |
| YDL226C | | GCS1 | NAT | deletion | no |
| YDL232W | | OST4 | NAT | deletion | no |
| YDR027C | | VPS54 | NAT | deletion | no |
| YDR056C | | YDR056C | NAT | deletion | yes |
| YDR304C | | CPR5 | NAT | deletion | yes |
| YEL002C | | WBP1 | NAT | DAmP | no |
| YER004W | | FMP52 | NAT | deletion | yes |
| YER019C-A | | SBH2 | NAT | deletion | yes |
| YER053C-A | | YER053C-A | NAT | deletion | yes |
| YER083C | | RMD7 | NAT | deletion | yes |
| YGR036C | | CAX4 | NAT | deletion | yes |
| YGR105W | | VMA21 | NAT | deletion | no |
| YHR007C | | ERG11 | NAT | DAmP | no |
| YHR060W | | VMA22 | NAT | deletion | no |
| YIL005W | | EPS1 | NAT | deletion | yes |
| YIL040W | | APQ12 | NAT | deletion | yes |
| YIL109C | | SEC24 | NAT | DAmP | no |
| YJL029C | | VPS53 | NAT | deletion | no |
| YJL073W | | JEM1 | NAT | deletion | yes |
| YLR035C-A | | YLR035C-A | NAT | deletion | no |
| YLR056W | | ERG3 | NAT | deletion | no |
| YLR268W | | SEC22 | NAT | deletion | no |
| YLR372W | | SUR4 | NAT | deletion | no |
| YML013W | | SEL1 | NAT | deletion | yes |
| YML125C | | YML125C | NAT | DAmP | no |
| YML128C | | MSC1 | NAT | deletion | no |
| YMR202W | | ERG2 | NAT | deletion | no |
| YMR296C | | LCB1 | NAT | DAmP | no |
| YNL085W | | MKT1 | NAT | deletion | yes |
| YNL125C | | ESBP6 | NAT | deletion | yes |
| YNL323W | | LEM3 | NAT | deletion | yes |
| YNL327W | | EGT2 | NAT | deletion | yes |
| YPL094C | | SEC62 | NAT | DAmP | yes |
| YPR148C | | YPR148C | NAT | deletion | yes |
| YPR183W | | DPM1 | NAT | DAmP | no |
| YBR052C | | YBR052C | NAT | deletion | no |
| YBR132C | | AGP2 | NAT | deletion | no |
| YCR043C | | YCR043C | NAT | deletion | no |
| YDR086C | | SSS1 | NAT | DAmP | no |
| YEL015W | | EDC3 | NAT | deletion | no |
| YGL095C | | VPS45 | NAT | deletion | no |
| YGL098W | | USE1 | NAT | DAmP | no |
| YHR135C | | YCK1 | NAT | deletion | no |
| YIL016W | | SNL1 | NAT | deletion | no |
| YIL040W | | APQ12 | NAT | deletion | no |
| YIL105C | | SLM1 | NAT | deletion | no |
| YJL026W | | RNR2 | NAT | DAmP | no |
| YKL119C | | VPH2 | NAT | deletion | no |
| YLR130C | | ZRT2 | NAT | deletion | no |
| YLR347C | | KAP95 | NAT | DAmP | no |
| YMR183C | | SSO2 | NAT | deletion | yes |
| YNL085W | | MKT1 | NAT | deletion | no |
| YNL238W | | KEX2 | NAT | deletion | no |
|  | |  |  |  |  |
|  | |  |  |  |  |
| KAN-marked test strains: | | |  |  |  |
| Orf Name | | Gene Name | Marker | Mutation Type | Successfully remade? |
| YBR036C | | CSG2 | KAN | deletion | no |
| YBR058C-A | | TSC3 | KAN | deletion | no |
| YBR096W | | YBR096W | KAN | deletion | yes |
| YBR097W | | VPS15 | KAN | deletion | no |
| YBR106W | | PHO88 | KAN | deletion | yes |
| YBR132C | | AGP2 | KAN | deletion | no |
| YBR234C | | ARC40 | KAN | deletion | no |
| YBR255W | | YBR255W | KAN | deletion | yes |
| YCR017C | | CWH43 | KAN | deletion | yes |
| YCR034W | | FEN1 | KAN | deletion | yes |
| YDL072C | | YET3 | KAN | deletion | yes |
| YDL232W | | OST4 | KAN | deletion | no |
| YDR027C | | VPS54 | KAN | deletion | no |
| YDR032C | | PST2 | KAN | deletion | yes |
| YDR057W | | YOS9 | KAN | deletion | yes |
| YDR410C | | STE14 | KAN | deletion | no |
| YDR476C | | YDR476C | KAN | deletion | yes |
| YDR518W | | EUG1 | KAN | deletion | yes |
| YDR525W | | API2 | KAN | deletion | yes |
| YEL015W | | EDC3 | KAN | deletion | no |
| YEL022W | | GEA2 | KAN | deletion | yes |
| YER044C | | ERG28 | KAN | deletion | no |
| YER053C-A | | YER053C-A | KAN | deletion | yes |
| YER083C | | RMD7 | KAN | deletion | no |
| YER087C-B | | SBH1 | KAN | deletion | yes |
| YER100W | | UBC6 | KAN | deletion | no |
| YER151C | | UBP3 | KAN | deletion | no |
| YGL012W | | ERG4 | KAN | deletion | yes |
| YGL095C | | VPS45 | KAN | deletion | no |
| YGL200C | | EMP24 | KAN | deletion | yes |
| YGL223C | | YGL223C | KAN | deletion | no |
| YGR036C | | CAX4 | KAN | deletion | no |
| YGR086C | | PIL1 | KAN | deletion | yes |
| YGR105W | | VMA21 | KAN | deletion | yes |
| YGR130C | | YGR130C | KAN | deletion | yes |
| YGR157W | | CHO2 | KAN | deletion | no |
| YHR060W | | VMA22 | KAN | deletion | yes |
| YHR123W | | EPT1 | KAN | deletion | yes |
| YHR135C | | YCK1 | KAN | deletion | no |
| YIL016W | | SNL1 | KAN | deletion | no |
| YIL040W | | APQ12 | KAN | deletion | no |
| YIL076W | | SEC28 | KAN | deletion | no |
| YIL105C | | SLM1 | KAN | deletion | yes |
| YJL029C | | VPS53 | KAN | deletion | no |
| YJL168C | | SET2 | KAN | deletion | no |
| YJR118C | | ILM1 | KAN | deletion | no |
| YLR056W | | ERG3 | KAN | deletion | no |
| YLR130C | | ZRT2 | KAN | deletion | no |
| YLR372W | | SUR4 | KAN | deletion | yes |
| YLR396C | | VPS33 | KAN | deletion | no |
| YML013W | | SEL1 | KAN | deletion | no |
| YML101C | | CUE4 | KAN | deletion | yes |
| YML128C | | MSC1 | KAN | deletion | no |
| YMR119W | | ASI1 | KAN | deletion | yes |
| YNL085W | | MKT1 | KAN | deletion | yes |
| YNL125C | | ESBP6 | KAN | deletion | no |
| YNL280C | | ERG24 | KAN | deletion | no |
| YOR036W | | PEP12 | KAN | deletion | yes |
| YOR198C | | BFR1 | KAN | deletion | no |
| YPR135W | | CTF4 | KAN | deletion | no |
| YPR173C | | VPS4 | KAN | deletion | no |
