# Supplementary material for: A strategy for extracting and analyzing large-scale quantitative epistatic interaction data
Source: Genome Biol. 2006 Jul 21;7(7):R63. doi: 10.1186/gb-2006-7-7-r63 (PMC1779568; doi:10.1186/gb-2006-7-7-r63)
Supplement: Additional data file 3 — Image capturing setup used for data collection. [file gb-2006-7-7-r63-S3.pdf]

## Additional File 3 - Image Capture Setup

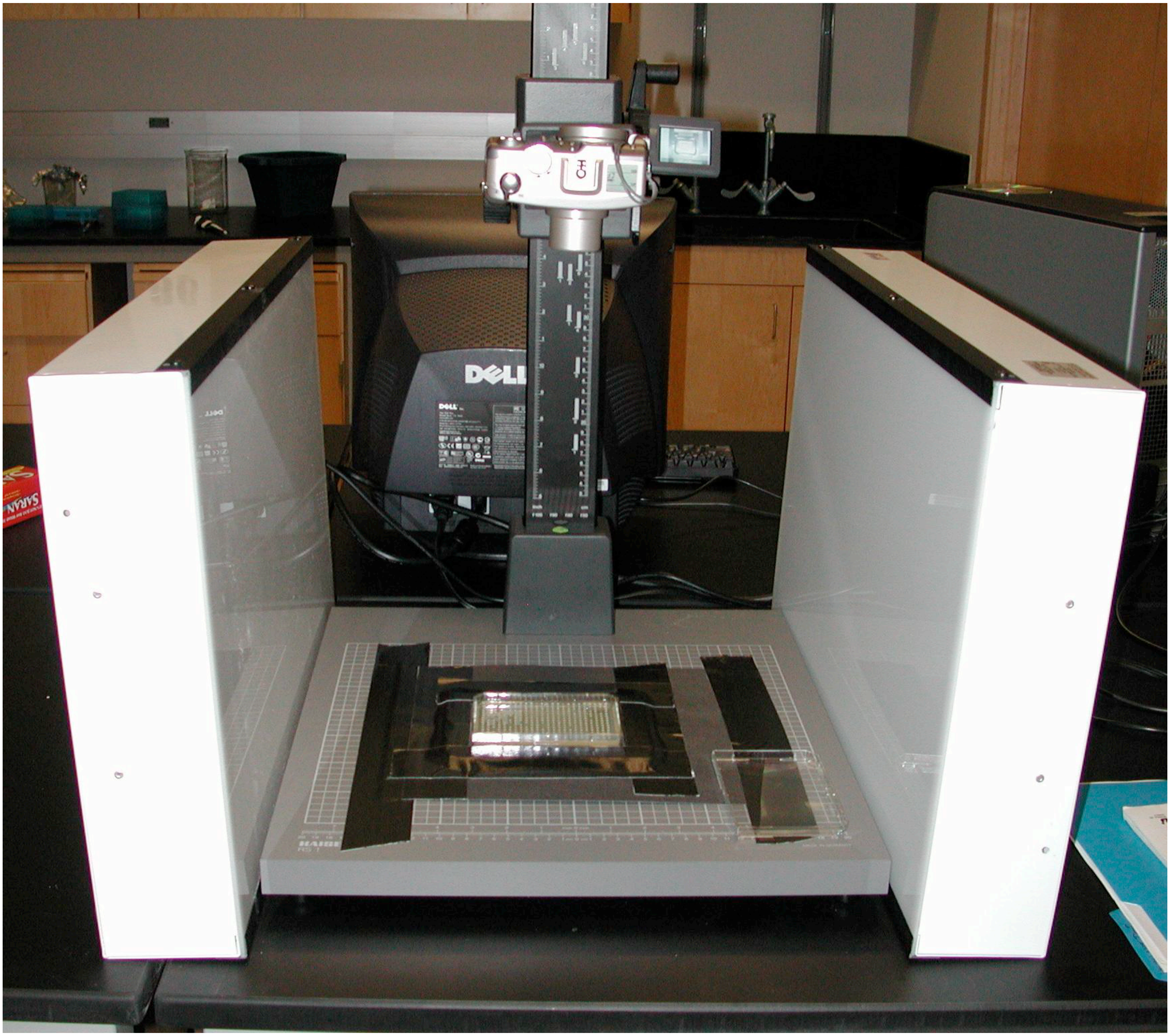


Shown are the digital camera, camera stand, and lighting sources assembled for image capturing.
