# Supplementary material for: A strategy for extracting and analyzing large-scale quantitative epistatic interaction data
Source: Genome Biol. 2006 Jul 21;7(7):R63. doi: 10.1186/gb-2006-7-7-r63 (PMC1779568; doi:10.1186/gb-2006-7-7-r63)
Supplement: Additional data file 9 — Explanation of the data included in Additional data files 4, 5, 6, 7, 8. [file gb-2006-7-7-r63-S9.doc]

The colony size data is given as a formatted text file with the following format:

The data for the NAT marked strain with a deletion of ORF YAL001W would be given as

NAT|YAL001W

KAN|1|1|ORFNAME_A|SIZE_A1|SIZE_A2|SIZE_A3|SIZE_A4|SIZE_A5|SIZE_A6

KAN|1|2|ORFNAME_B|SIZE_B1|SIZE_B2|SIZE_B3|SIZE_B4|SIZE_B5|SIZE_B6

...

where the first two numbers in the rows beginning with KAN indicate the row and column coordinates of the particular KAN-marked strain on the physical experimental plate. The sizes following ORFNAME_A indicate the replicate measurements for the double mutant with deletions of YAL001W and ORFNAME_A. The sizes are in pairs that come from the same experimental plate (SIZE_A1 and SIZE_A2 come from measurements of adjacent colonies on the same plate, whereas SIZE_A3 comes from a different plate).

An example experimental plate is shown below:


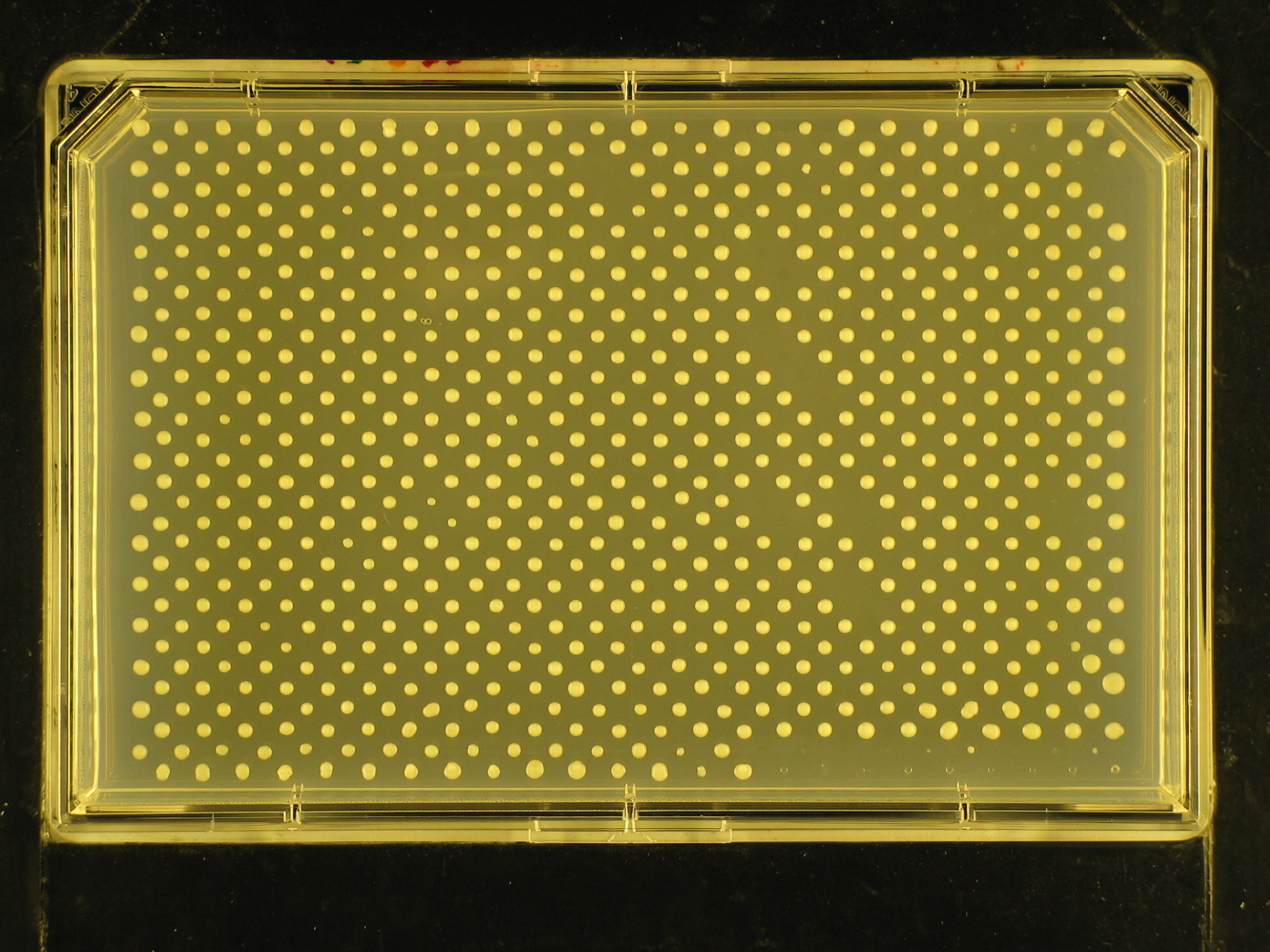


Replicate colonies are arranged diagonally adjacent to each other.

Five files containing colony sizes are included. These correspond to 4 data sets that were processed separately (two consisting of constitutive hypomorphs of essential genes and two consisting of deletions of nonessential genes) as well one data set of replicate measurements of single mutants generated by crossing a NAT-marked wild-type strain to the library of 384 KAN-marked strains.
